# Current Understanding of Feather Keratin and Keratinase and Their Applications in Biotechnology

**DOI:** 10.1155/bri/6619273

**Published:** 2025-04-22

**Authors:** Thanakorn Moktip, Lakha Salaipeth, Ana Eusebio Cope, Mohammad J. Taherzadeh, Takashi Watanabe, Paripok Phitsuwan

**Affiliations:** ^1^LigniTech-Lignin Technology Research Group, School of Bioresources and Technology, King Mongkut's University of Technology Thonburi, Bangkuntien, Bangkok 10150, Thailand; ^2^Division of Biochemical Technology, School of Bioresources and Technology, King Mongkut's University of Technology Thonburi, Bangkuntien, Bangkok 10150, Thailand; ^3^Natural Resource Management and Sustainability, School of Bioresources and Technology, King Mongkut's University of Technology Thonburi, Bangkuntien, Bangkok 10150, Thailand; ^4^Future Genetic Resources Cluster, Rice Breeding Innovation Platform, IRRI, Los Banos, Philippines; ^5^Swedish Centre for Resource Recovery, University of Borås, Borås 50190, Sweden; ^6^Research Institute for Sustainable Humanosphere, Kyoto University, Kyoto 611-0011, Japan

**Keywords:** bioactive peptide, chicken feather, keratin degradation, keratinase, protease

## Abstract

The food industry generates substantial keratin waste, particularly chicken feathers, which are rich in amino acids and essential nutrients. However, the insolubility of keratin presents a significant challenge to its conversion. Keratinase, an enzyme produced by certain fungi and bacteria, offers a promising solution by degrading feather keratin into amino acids and soluble proteins. Among these, bacterial keratinase is notable for its superior stability and activity, although its production remains constrained, necessitating continued research to identify efficient microbial strains. Keratin-derived hydrolyzates, recognized for their biological and immunological properties, have garnered significant research interest. This review examines the structural characteristics of chicken feather keratin, its resistance to conventional proteases, and advances in keratinase production and purification techniques. Additionally, the keratin degradation mechanism and the adoption of environmentally friendly technologies for managing feather waste are explored. Finally, the review highlights the potential applications of keratinase across diverse industries, including animal feed and cosmetics.

**Summary**

• Food industry produces keratin waste like feathers, a source of amino acids and peptides.
• Ongoing research seeks robust keratinase-producing strains to improve productivity.
• Keratin hydrolyzates with biological activities spark interest in production.
• Green technology manages chicken feather waste sustainably to marketable products.

## 1. Introduction

Keratin, a protein-rich material, possesses a complex physiochemical structure. Based on secondary structures and amino acid composition within polypeptides, keratins can be categorized into α- and β-keratins, which consist of α-helical coils and β-pleated sheets as majorities in the structures, respectively. Keratin can also be classified as soft and hard based on the number of cysteine residues [1]. Soft keratin, with <10% cysteine, is present in the epidermis of skin, while hard keratin, containing around 10%–14% cysteine, can be found in hair, nails, feathers, and claws [2].

The structural rigidity of keratin arises from its supramolecular organization, wherein polypeptide chains form a structure through hydrogen bonds, hydrophobic interactions, and disulfide bonds. Cysteine residues form intra- or intermolecular disulfide bonds, which contribute to compacting the keratin structure and enhancing the structural stability [1, 3]. α-Keratin is commonly present in mammals, while β-keratin is found in avian and reptilian tissues [4]. Examples of α-keratin include wool, hair, and horns, whereas those of β-keratin are scales, claws, beaks, and feathers [5]. Furthermore, α-keratin has been reported to be more resistant to degradation compared to β-keratin because α-keratin has more disulfide linkages [6]. A vast amount of keratinous side product is generated from animal production and meat-related industries, and the accumulation of antlers, bristles, claws, hair, hooves, horns, wool, and feathers deteriorates the environment [7].

These days, the poultry industry has grown rapidly due to the high demand for poultry meat products. In 2020, approximately 100.5 million tons of the poultry meat was produced worldwide, together with the generation of solid waste [7]. Among the massive solid waste, over 4.7 million tons consist of chicken feathers, which pose a challenge for waste management [8]. The disposal of feather waste in landfills has detrimental effects on the environment and human health. These negative impacts include the nitrate leaching into groundwater, phosphorus release into water body, bad odors associated with the waste dump, and accumulation of pathogens [7, 8].

While incinerating feather waste offers a solution to managing the massive waste, it emits air pollutants and toxic gases, such as ammonia, nitrous oxide, hydrogen sulfide, carbon dioxide, and methane, which contributes to global warming. Thus, air emission needs purification or treatment prior to release into the environment, rendering this approach a costly process [9]. In alignment with the sustainable development goals (SDG), which target solutions enabling economic and societal development without environmental damage, a more eco-friendly approach to managing feather waste, emphasizing environmental preservation, becomes imperative [10–12].

Indeed, chicken feather contains more than 85% crude protein, 70% amino acids, minerals, vitamins, and growth factors, rendering them a compelling raw material for the biotechnological production of supplements, feed, cosmetics, fertilizer, and more [13]. Furthermore, it is a keratin-rich material (90%), which has a high potential to be applied in biomaterials [14, 15]. However, the limitation of using chicken feathers is the natural resistance to degradation. This recalcitrance arises from the keratin structure, which is formed through the combination of hydrogen bonds, hydrophobic forces, and, importantly, the disulfide bonds connecting internal and inter-polypeptide chains. This bonding results in a dense, rigid polymeric structure. This structural organization makes keratin in chicken feathers very stable and has a high mechanical strength. Efforts have been undertaken to extract value from chicken feathers through the utilization of chemical and physical methods [16]. These methods include alkaline extraction, oxidation, reduction, sulfitolysis, ionic liquids, steam flash explosion, microwave radiation, and thermal hydrolysis process [17].

A common strategy to utilize chicken feathers through these methods involves producing feather meal. The feather was heated under pressure and ground to powder. This milled feather powder was supplemented with ruminant feed. However, the high number of disulfide bonds within the feather impedes the enzymatic degradation, leading to poor digestion and very minimal release of amino acids and soluble protein [7]. Furthermore, the application of high temperature and pressure leads to the degradation of essential amino acids, such as tryptophan, methionine, and lysine. As a consequence, the resulting amino acid quantity diminishes, leading to a reduction in available nutrients [18]. Another common method for treating chicken feathers is alkaline hydrolysis. While effective in enhancing keratin digestibility, alkaline hydrolysis can result in modification or degradation of amino acids.

Alkaline hydrolysis of keratins at higher temperatures leads to the degradation of the thermally unstable amino acids, asparagine, glutamine, arginine, serine, threonine, and cysteine. For example, alkaline conditions with heat (up to 170°C) induce the formation of non-nutritive amino acids, such as lysinoalanine, lanthionine, and 8-aminoalanine [17, 19, 20], thereby decreasing the value of resulting hydrolyzates for applications. Thus, proper management of feather waste is necessary to maximize its value while minimizing its environmental impact.

In recent years, there has been considerable interest in biological solutions that utilize enzymes and microorganisms for the decomposing of chicken feathers. This is attributed to enzymatic and microbiological procedures that maintain the activity of degradation products, while also being ecologically beneficial as they can be performed under mild conditions with less toxic waste generation [13, 21].

Keratinases (EC 3.4.21/24/99.11) are a class of proteolytic enzymes capable of breaking down keratinous biomass, resulting in high-nutrient protein hydrolyzates [21]. These enzymes are found in various fungi and bacteria such as *Candida*, *Aspergillus*, *Streptomyces*, and *Bacillus* [22, 23]. *Bacillus* is the most investigated genus for keratinase-producing bacteria that can degrade chicken feather keratin [24–27] and multiple *Bacillus* species showing potential for commercial-scale keratinase production [28]. For example, *B. subtilis* strains have been identified as excellent keratin degraders within chicken feathers. Moreover, *B. subtilis* has been verified as a safe host bacterium for the synthesis of industrial enzymes, vaccine antigens, and medicines [23]. Moreover, specific strains of *B. pumilus* exhibit good ability to degrade both ɑ- and β-keratins, showing the potential for enzymatic processing of wool and feather toward waste biorefinery [29].

Furthermore, the primary constituents of hydrolysis products resulting from keratin degradation by keratinases are amino acids and soluble peptides. These peptides have been shown to have antioxidant properties, angiotensin I–converting enzyme (ACE) inhibitory, dipeptidyl peptidase IV (DPP IV) inhibitory, antifungal, and antibacterial activity [17, 18, 30–33]. Therefore, utilizing keratinolytic microorganisms and their enzymes seems to offer a sustainable approach for managing keratin-rich waste. This approach not only diminishes environmental impacts but also increase opportunity to valorize feather waste to produce useful chemicals.

The biochemical property of keratinase, along with its functional roles in keratin waste degradation and enzyme production for industrial use, has been the subject of some recent reviews, providing useful information [7, 13, 18, 21, 34]. This review, written from the perspectives of green technology and biotechnological applications, aims to shed light on the conversion of keratin to marketable products using biological approaches. The exploration starts with an examination of keratin's structure, microbial sources, key enzymes involved in keratin degradation, and techniques for purifying enzymes. Furthermore, we delve into the potential applications of keratinase or keratinolytic microorganisms across various industries, including detergents, agriculture, wastewater treatment, leather, textile, biofuel, and feed industries. Additionally, we explore the utilization of keratin-rich hydrolyzate as an active ingredient within the realms of pharmaceuticals and cosmetics.

## 2. Structure of Keratin in Chicken Feathers

Keratins are fibrous proteins, categorized into two types: α-keratin and β-keratin determined by their secondary protein structures [4]. These secondary structures are formed due to the difference in polypeptide sequences, comprising different polar and charged amino acids of various sizes. The intra- and interactions among amino acids within the polypeptide chains turn into helix or line up with each other, forming sheet-like structures, ultimately yielding α-helix and β-pleated sheet structures, respectively [35].

**Figure 1:**
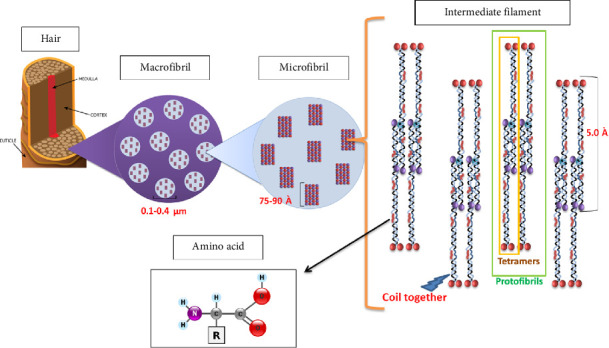
α-Keratin structure in mammalian hair. The formation of α-keratin structure in mammalian hair stems from the organization of hair macrofibrils, which are formed from tightly packed bundles of microfibrils within the hair cortex. Each microfibril contains intermediate filaments that are composed of protofibrils. Protofibrils arise from the assembly of two tetramers; each tetramer consists of two heterodimers. A heterodimer is formed by the parallel alignment of one Type I keratin and one Type II keratin monomer. These heterodimers further assemble into tetramers through four possible configurations. The heterodimer originates from keratin monomers, each of which has three distinct regions: the head domain (N-terminal domain), the rod domain, and the tail domain (C-terminal domain). The rod domain consists of four right-handed α-helical subdomains separated by nonhelical β-turns known as “linker” regions. Specific hydrophobic and charged amino acids at particular positions within the α-helical subdomains contribute to stabilizing the α-helix structure. The aggregated structure is further stabilized through hydrogen and disulfide bonds, creating a robust and cross-linked keratin network (modified with permission from Qiu et al. [34]).

α-Keratin constitutes the primary keratin present in mammalian fibers and has a molecular mass of approximately 60–80 kDa. Mammalian epidermal components such as hair, nails, wool, hooves, and horns are instances of materials containing α-keratin. α-Helical coils, categorized as Type I (acidic) and Type II (basic/neutral) protein chains, are coiled together to form elongated α-helix filaments that form fibrils by interchain or intrachain bonding [19, 33] (Figure 1).

The β-keratin is constructed from β-strands. Generally, β-keratin has a molecular mass of approximately 10–22 kDa, and it is typically found in avian and reptiles [2, 35]. Examples of tissues containing β-keratin components encompass scales, claws, beaks, and feathers.

Within chicken feathers, keratin acts as a structural protein originating from vertebrate epithelia, playing multiple physiological roles. It serves as a water-repelling agent, acts as a barrier against infections, and functions as a cushion against mechanical impacts. The structural keratins within chicken feathers include α-helix, β-pleated sheet, and a limited number of disordered structures, where β-keratin represents a major component [19, 36]. Four β-strands or monomeric structure keratin is packed, forms parallel or antiparallel, and is held together by hydrophobic interaction with another monomeric structure keratin to form a dimeric structure.

**Figure 2:**
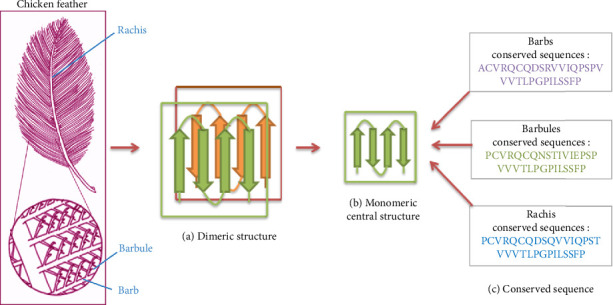
β-Keratin in feather structure. Dimeric (β-sandwich) structure of β-keratin (a) was formed by the antiparallel organization of the four strands of monomeric structures (b). The structural integrity of β-keratin differed across barbs, barbules, and rachis based on the amino acid composition (c). The fundamental structure of β-keratin is a four-strand β-sheet, referred to as the monomeric structure. Two monomeric structures align in reverse stacking to form a dimer through hydrophobic face-to-face interactions. Within this dimeric structure, salt bridges, hydrogen bonds, and disulfide bonds are established between specific amino acids of the two monomers. The assembly continues with a head-to-tail arrangement of four dimers, resulting in the formation of a left-handed helix. This β-keratin filament features four repeating units per turn, achieved through hydrogen and disulfide bonding, with the progressive rotation of piled dimers at approximately 45°. Compared to α-keratin, which is characterized by a coiled-coil α-helical structure with distinct head, rod, and tail domains, β-keratin adopts a β-sheet structure and forms filaments through a combination of stacking and helical assembly. Additionally, the interactions in β-keratin filaments are predominantly mediated by hydrophobic interactions and secondary bonds, while α-keratin relies heavily on coiled-coil interactions within its helical domains for structural integrity (modified with permission from Qiu et al. [34]).

Several bonds, such as hydrogen bonds, disulfide bonds, and hydrophobic interactions involving amino acids, are formed within the dimeric structure (Figure 2). Through hydrogen and disulfide bonding, the helical structure transforms into a left-handed helix, featuring four repeating units per turn [34].

The structure of chicken feathers comprises barbules, barbs, and a rachis, as depicted in Figure 2. Barbs emerge from the rachis, and barb connects to the barbules. Protein variation and structure can be linked to structural changes in various feather portions.

On average, whole feathers contain 32.2% of α-helix, 53.6% of β-sheets, and random coils, while turns account for 14.2% [37]. Indeed, the barb and barbules fraction exhibits slightly higher proportion of α-helix compared to β-sheet structure, while the rachis has more β-sheet than α-helix structure [38]. Furthermore, chicken feathers are abundant in arginine, serine, proline, valine, leucine, threonine, glutamate, glycine, aspartate, histidine, lysine, and methionine among other amino acids [39] (Table 1). Each amino acid causes bonding within keratin.

For example, a disulfide bond is established between cysteine–cysteine pairs, while glycine facilitates hydrophobic interactions. Glutamine–serine and aspartic acid–lysine form hydrogen bonds. Notably, the disulfide bond is perceived as a particularly challenging linkage, as it contributes to rigidity and proves challenging to cleave. Therefore, the disulfide bond content has been employed to determine whether the keratin is soft or hard [34, 40].

In addition to disulfide linkages, β-keratin has been reported to contain a high quantity of nonpolar amino acids, particularly glycine. Indeed, the keratin structures of barbules, barbs, and rachis differ in their glycine quantity and composition, with rachis containing the highest glycine [41–43]. This evidence suggests that hydrophobic interactions play an essential role in structural stiffness, elucidating why rachis exhibits greater hydrophobic and resistant to degradation compared to barb and barbule [34]. An exemplar of this phenomenon is the degradation of turtle scutes, which pose a more challenging disintegration process compared to chicken feathers due to their higher glycine content [34, 44]. Consequently, the presence of a substantial amount of cysteine and the nonpolar amino acid glycine emerge as a key factor in conferring resistance to keratin degradation.

## 3. Chicken Feather Conversion

### 3.1. Thermochemical Methods

Various techniques for transforming chicken feathers into value-added products have been demonstrated, with notable emphasis on thermochemical methods for the extraction of keratins or the production of peptides and amino acids. Usually, thermochemical methods rely on the solubility of keratin at moderate to high temperatures, often involving organic solvents, hydrothermal procedures, hydrogen, and disulfide bond oxidation or reduction, as well as hydrolysis through alkali or acid treatment.

Among these techniques, the reduction process emerges as a prevalent method for keratin extraction from chicken feathers [45–49] (Table 2). Furthermore, the utilization of green solvents, such as ionic liquids and deep eutectic solvents, has been applied for keratin extraction. These solvent applications are under thorough investigation to comprehend their mechanisms in the process of extracting keratin from chicken feathers.

Frequently employed thermochemical techniques for keratin extraction commonly involve the use of reducing agents, such as 2-mercaptoethanol, sulfites, and bisulfite. These agents facilitate the liberation of cysteine residues, leading to the acquisition of soluble keratins (Table 2).

This reduction process cleaves disulfide linkages within the keratin structure, resulting in cysteine thiol (keratin-Cys-S or reduced keratin); and cysteine-S-sulfonate residue (Keratin-Cys-SSO-3 or Bunte salt) [48]. Methods for precipitating keratin after solubilization require substantial volumes of solvents, such as acetone, to transform soluble keratin into keratin powder. Due to the environmental impact of this acetone, its recycling or proper disposal is necessary.

Ionic liquids provide an alternative for keratin treatment. Ionic liquids such as imidazole ionic liquids (e.g., [Bmim]Cl, [Amim]Cl, [Bmim]Br) could extract keratin from poultry feathers in a short amount of time. This method shows promise in recovering high-value keratin, nonvolatility, easy recycling, and no pollutant discharge [14, 55]. For example, when extracting keratin from duck feathers using [Bmim]Cl in combination with Na_2_SO_3_ at 90°C for 60 min, a dissolution rate of 96.7% was achieved. The soluble keratin was subsequently precipitated with water, resulting in a keratin yield of 75.1% keratin [14].

In contrast, extraction of keratin from chicken feathers utilized hydrophobic ionic liquid (IL) ratio of 1-hydroxyethyl-3-methylimidazolium bis(trifluoromethanesulfonyl)amide ([HOEMIm][NTf2]) and NaHSO_3_ at 1:1. The solid (feathers) to ionic liquid ratio was 1:40, and the process was carried out at 80°C for 4 h, resulting in a 21% keratin yield [52]. This approach is convenient as ([HOEMIm][NTf2]) can be readily recovered using water owing to its hydrophobicity. Furthermore, this ionic liquid can be reused up to five times [52]. However, extraction parameters such as temperature, ionic liquid type, and keratin type (e.g., feather, hair) need adjustment, as they have been shown to impact keratin yield. Furthermore, although ionic liquids proved to be a viable option for treating keratin waste, they incurred higher costs compared to conventional solvents. Additionally, the application technique was more intricate and complex.

It is important to remember that the goal of keratin extraction is to break disulfide bonds and alter the keratin structure, enabling its solubilization. It has been noted that hydrothermal treatment involving alkalis and acids can be employed for this purpose. This process is typically conducted at high temperature (80°C–140°C) or under high steam pressure (10-15 psi) to enhance extraction performance [36, 56]. Although alkalis possess effective protein-solubilizing capabilities, they are avoided in the keratin extraction process. This caution arises from the fact that certain amino acids, such as asparagine, glutamine, arginine, serine, threonine, and cysteine, are degraded by the alkalis at high temperature, when exposed to alkalis, ultimately leading to amino acid losses [57]. For example, the addition of alkali to the reactions led to the degradation of methionine, lysine, and tryptophan [19], Also, glutamine or asparagine was easily degraded at high temperature [58, 59]. Cysteine residues formed lanthionine, a toxic substance, under weak alkaline treatment [60].

Furthermore, variations in the effects of heating processes on amino acids have been documented when alkalis are present. For example, the application of microwave-generated heat in alkali-based keratin extraction better preserved essential amino acids (leucine and valine) and nonessential amino acids (serine, glycine, and tyrosine) compared to autoclaving [36]. Therefore, the heating process should be considered when alkalis are applied in the keratin extraction process.

Sulfuric acid is frequently employed in keratin extraction, a process referred to as acid hydrolysis. Numerous studies are exploring various conditions for keratin hydrolysis by employing different types of acids (e.g., sulfuric, nitric, and phosphoric acids), concentrations, temperatures, and solid-to-liquid ratios. These investigations aim to yield a liquid fraction containing keratins and related peptides. This process holds promise for industrial applications due to the cost-effectiveness and ease of handling of acid [61]. However, the majority of acid treatment methods involve high temperatures. For example, applying sulfuric acid in keratin hydrolysis resulted in 88.83% crude protein yield after a 5-h treatment at 90°C [61]. However, this approach led to the degradation of specific amino acids and their transformation into hazardous compounds.

To improve extraction, preserve keratin yield, and prevent amino acid loss, some studies have demonstrated the addition of ionic liquids such as 1-butyl-3-methylimidazolium chloride ([Bmim]Cl) to hydrochloric acid under ultrasonic irradiation [62]. Research revealed that through the utilization of [Bmim]Cl in conjunction with ultrasonication, the requirement for hydrochloric acid could be minimized to as low as 8 mol/L. This procedure was conducted at 80°C, with a short treatment time of 1.4 h, and yielded 83.1% keratin. The liquid fraction contained peptides, resulting in lower amino acid generation [63]. This study is promising in terms of low acid concentration used, short operation time, and low-energy input, as the hydrolysis process of feather keratin at the industrial level used 12 mol/L hydrochloric acid at 110°C for 12 h [63].

### 3.2. Biological Methods

#### 3.2.1. Microbial Treatment

Indeed, keratin wastes are capable of undergoing natural decomposition. However, the degradation process takes several weeks. Thus, there is a dedicated pursuit to identify keratinolytic microorganisms that can effectively expedite the degradation of keratin wastes [26]. Keratinolytic microorganisms are recognized for generating specific proteases to degrade keratins.  Table 3 shows a list of microorganisms that produce keratinase enzymes. Keratinases are distributed in bacteria [64, 87–89] and fungi [90, 91].

The predominant research emphasis is on bacteria for keratin waste decomposition, primarily owing to their rapid enzyme production, rapid proliferation, applicability in industrial contexts, and the potential to utilize the bacteria as a whole for keratin degradation, thus obviating the need for enzyme extraction.

*Chryseobacterium* sp., *B. licheniformis*, *Arthrobacter* sp., *Stenotrophomonas* sp., *B. pumilus*, and *B. thuringiensis* are among the bacteria that produce keratinase enzyme (Table 3). *Bacillus* species have been identified in larger quantities compared to other species. However, keratinolytic bacteria have widely varying characteristics that are influenced by factors such as isolation method, bacterial strain, and environmental conditions (pH, temperature) (Table 3).

Keratin-rich sources, such as soil from chicken waste, animal waste, and slaughterhouses, are suitable for isolating keratin-degrading bacteria (Table 3). A method to identify keratinase-producing bacteria involves assessing their ability to degrade keratin or a similar protein substrate, such as casein or skim milk. Subsequently, bacteria are often identified following isolation by 16S rRNA gene sequencing [64].

*B. licheniformis* appears to be a potential bacterium for keratin degradation [65, 92, 93]. It also produces stable enzymes that can tolerate a wide range of pH and temperatures [65]. Actually, keratin substrate is ideal for the induction of keratinase production [94]. However, some bacteria do not require keratin substrate for inducible keratinase production, as they possess a constitutive enzyme system [1, 94]. It is noteworthy to mention that while several microorganisms have been identified as keratinase producers, most well-characterized keratinolytic microorganisms exhibit the capacity to hydrolyze β-keratin found in chicken feathers.

Only a limited number of bacteria, such as *B. subtilis*, *B. pumilus*, and *Stenotrophomonas*, have been documented to hydrolyze both α- and β-keratins [29, 95]. This might reflect that β-keratin is more prone to the enzymatic degradation. Furthermore, the choice of substrate for screening keratinolytic microorganisms is another factor that restricts the opportunity to discover novel keratinolytic bacteria with unique enzymatic functions. This tendency arises from the frequent utilization of β-keratin-rich chicken feathers as screening substrates in numerous studies. This choice is driven by the feathers' rapid and observable deconstruction, which takes place in a short span of time. Hence, there is a need to devise additional screening strategies.

#### 3.2.2. Chicken Feather Degradation by Enzymes

Due to its high number of hydrogen bonds and disulfide bridges between chains, keratin is resistant to degradation by common protease enzymes such as pepsin, trypsin, and papain [61]. Keratin degradation through keratinase is currently a subject of intense investigation, with researchers approaching a comprehensive understanding of the underlying mechanism [34].

Keratinase is the primary enzyme responsible for the degradation of chicken feathers. They are peptide hydrolases that catalyze the hydrolysis of simple peptide bonds in keratin [34]. Keratinase has been categorized as a member of the MEROPS protease family, a classification determined by its catalytic mechanism or substrate specificity.

**Figure 3:**
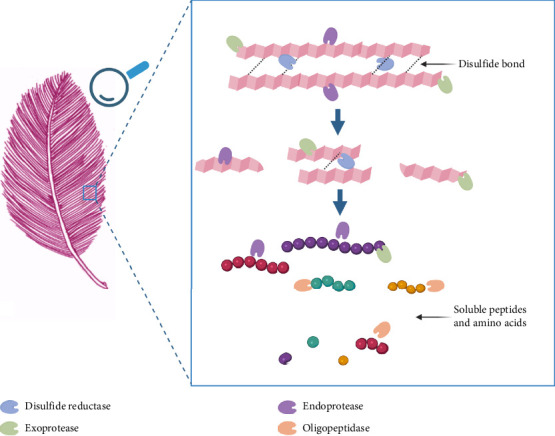
Possible enzymatic hydrolysis of keratin in chicken feather. The enzymatic hydrolysis of keratin involves a coordinated action of multiple enzymes. Disulfide reductases cleave disulfide bonds, allowing the cross-linked polypeptide chains to undergo denaturation. Exoproteases target the terminal ends of polypeptide chains, while endoproteases cleave peptide bonds within the internal regions of these chains. The oligopeptides generated by the combined actions of exo- and endoproteases are further broken down by oligopeptidases, resulting in the release of short peptides and free amino acids.

Proteases such as endoprotease, exoprotease, oligoprotease, and, importantly, enzymes or chemicals that break the disulfide bond are required for complete keratin degradation to amino acids or soluble peptides (Figure 3) [13, 28]. The process of keratin degradation is thought to involve two key steps: sulfitolysis and proteolysis [11]. Sulfitolysis of disulfide bonds within the keratin structure is a nonenzymatic process driven by sulfite ions, whose production is indirectly facilitated by cysteine dioxygenase through the oxidative catabolism of cysteine. In parallel, enzymatic reduction of disulfide bonds is catalyzed by disulfide reductases, collectively contributing to the disruption of keratin's structural rigidity. Disulfide bonds are broken, enabling exoproteases, endoproteases, and oligopeptidases to access polypeptide chains for hydrolysis into amino acids or soluble peptides (Figure 3). In the subsequent discussion, we address several aspects highlighted in a recent review by Qiu et al. [34]. This review delves deeply into how the utilization of amino acid sequences and conserved domains within MEROPS classification system can be employed to categorize a wide range of keratinases.

##### 3.2.2.1. Endoprotease

Endoprotease can break down peptide bonds within polypeptide chains. Endoprotease encompasses families such as S1, S8, S16, M4, M16, and M36. Within these families, S1, S8, and S16 are serine proteases, while M4, M16, and M36 are classified as metalloproteases, according to the MEROPS classification.

Among serine endoproteases, the S1 family represents the largest serine protease family. S1 peptidases have a catalytic triad of His, Asp, and Ser within their active sites. All S1 enzymes are endoacting, functioning through a mechanism involving nucleophilic attack and an acid hydrolysis [19]. *Streptomyces fradiae* [88], *Nocardiopsis* sp. [96], *Paenarthrobacter nicotinovorans* [1], and *Actinomadura viridilutea* [97] are among the microbial species that produce S1 endoproteases with keratinolytic activity. The majority of enzymes are active at alkali pH and high temperatures [98, 99].

While S8 protease exhibits some similarities in the catalytic triad mechanism to the S1 proteases, the arrangement of catalytic amino acids in their active sites is arranged differently: Asp, His, and Ser in the sequence [19]. *B. licheniformis* [65], *B. pumilus* [24, 29, 100], *B. cereus* [101], and *Thermoactinomyces* sp. [83] are among the bacteria that contain the majority of these enzymes. These enzymes function as endopeptidases, which are active at neutral to mild alkaline pH and thermostable [65].

A vast majority of S8 enzymes hydrolyze upon interaction with a hydrophobic residue within the keratin substrate, rendering them nonspecific in their action [19]. Despite distinctiveness in their sequences, members of both S1 and S8 families have the same evolutionary route and catalyze the same mechanism. In several members of these families, the presence of two calcium-binding sites in several members of these families aids in the development of thermal stability [29].

Unlike the S1 and S8 protease families, the S16 family is unique. Enzymes within the S16 family feature a Ser-Lys catalytic active site. *Fervidobacterium islandicum* hosts the S16 enzymes [34, 102]. The information about the S16 family remains unclear, necessitating further research to elucidate its characteristics.

Among metalloproteases, M4 protease family is classified as the zinc metalloproteases, due to the existence of a consensus zinc-binding HELTH motif. In this motif, a catalytic zinc ion is tetrahedrally coordinated within the active site by a histidine and glutamate from a HEXXH motif, as well as another glutamate residue and a water molecule. Example of proteases from M4 family includes GEOker, an enzyme produced by a thermophilic bacterium *Geobacillus stearothermophilus* AD-11 [103]. The catalytic triad of M4 family members, His372, Glu373, and His376, plays a crucial role in the enzymatic process of the GEOker and its activity on various substrate proteins. While the M4 protease family has been known for a considerable period, there has been no investigation into its specific function.

Another metalloendopeptidase belongs to M16 protease family. It has been documented that *Fervidobacterium islandicum* produces the M16 protease family [102]. Through the application of the real-time PCR technique, Kang et al. [102] showed substantial expression of at least four metalloproteases and two peptidases, indicating their prominent involvement in feather degradation. Based on gene annotations, one of the metalloproteases belonged to the insulin (M16) family. Compared to the crude enzyme alone, the addition of the recombinant M16 enzyme to the *F. islandicum* led to an approximately 1.5-fold increase in the degradation of native feathers [102]. However, biochemical, structural property, and catalytic mechanism of the certain enzymes were not subjected to further investigation. This underscores the need for additional research to delve into the precise operational mechanism of M16 keratinolytic proteases.

M36 peptidase family has been exclusively found in fungi. *Aspergillus fumigatus* is an example of M36 metalloendopeptidase producers. This enzyme family is also referred to as the fungalysin family [104]. Fungalysins represent a one-of-a-kind family of zinc-dependent peptidases that share low sequence similarity with the known bacterial peptidases of the thermolysin family. Moreover, the M36 protease family belongs to is categorized within the metallopeptidase clan MA(E), also known a commonly referred to as the gluzincin clan. This clan comprises metallopeptidases with a zinc ion linked by two histidines inside the motif His-Glu-Xaa-Xaa-His (HEXXH) [105, 106].

##### 3.2.2.2. Exoprotease

Exoprotease possesses the ability to cleave peptide bonds at the polypeptide chain's end. These enzymes fall within the serine protease category (S9 and S10), as well as the metalloprotease category (M14, M28, M38, and M55).

The catalytic function of S9 family serine proteases is executed by a catalytic triad in the order of Ser, Asp, and His. Proteases within this category include DPPV and DPPIV, which are secreted by a fungus *Trichophyton rubrum* [107]. Despite the S9 family proteases' effectiveness in attacking the chain end of keratin, their activity against keratin substrate is poor [107, 108]. Thus, it is believed that this enzyme could contribute to the degradation of keratin-derived hydrolysis products resulting from endopeptidase activity. That is, the S9 family might collaborate with an endoprotease to facilitate keratin degradation. Some research has shown that the collaborative action of endo- and exoproteases is crucial for the pathogenicity of dermatophytes during infection of keratinized tissues [109].

The S10 protease family employs a catalytic triad arranged as Ser, Asp, and His. The predominant function of most peptidases within this family is carboxypeptidase activity, involving the hydrolyze peptides from the C-terminal [108]. *T. rubrum* also secretes the S10 protease TruScpA (Ser238, Asp458, and His516) and TruScpB (Ser240, Asp459, and His517). Similar to the S9 protease family, the S10 protease family promotes and improves keratin degradation when used in conjunction with keratinolytic endoproteases [108].

M14 proteases function as carboxypeptidases, specializing in the removal of individual C-terminal amino acids from polypeptide chains. These enzymes recognize the free C-terminal carboxyl group, an important element of specificity. It has been reported that the production of *T. rubrum* protease (McpA), categorized in M14 protease family, was induced when keratin–soy was used as a nitrogen and carbon source [110]. His179, Glu182, and His309 serve as catalytic zinc ligands. Additionally, other M14A family amino acids, such as Arg237, Arg255, Tyr311, Tyr362, and Glu385, play a vital role in substrate binding and catalysis [111].

The M28 protease family has gained much research attention due to the enzyme in this family exhibiting high activity against different keratin-containing waste, including pig bristle and feather keratin [108]. This family is found in dermatophytes such as *T. rubrum* [107]. This protease contains the conserved motifs H147, D159, E194, D222, and H309, all of which involved in the interaction of two zinc atoms within this protease. The M28 protease Lap1 and Lap2 can hydrolyze peptides from the N-terminus to the X-pro or X-Ala motifs, which function as a stopping point [112]. Therefore, the M28 protease family might collaborate with endoprotease to improve the degradation rate of keratins.

*F. islandicum* secretes the M38 and M55 protease families [102], both of which play an important role in amino acid production. It is hypothesized that in the process of keratin degradation by *F. islandicum*, M38 protease may be associated in proteolysis, whereas M55 protease may be activated under starvation conditions and play a role in regulating the flow of peptides [34, 102].

##### 3.2.2.3. Oligopeptidase

Oligopeptidase is an enzyme that cleaves the short oligopeptides; however, it does not have any activity against proteins. This is because the enzyme's active site is positioned at the end of a narrow cavity, which only allows short peptides to pass through to it. That is, the keratin hydrolysis product (i.e., a short peptide) formed following endo- and exoprotease digestion becomes a substrate for oligopeptidase, Typically, oligopeptidase is found in the M3 and M32 protease families, among other families. Zinc-dependent metallopeptidases are found in the M3 and M32 protease families, both belonging to the MA (E) clan. Histidines are the two zinc ligands in the pattern His-Glu-Xaa-Xaa-His [113].

M3 oligopeptidases are intracellular oligopeptidases produced by bacteria, fungi, and mammals. The M3 protease family can only hydrolyze small peptides, which have been cleaved by endo- or exoproteases. As per the findings of this study [114], endoprotease (S8), exoprotease (M28), and oligopeptidase (M3) collaborate to improve keratin degradation. Within M32 protease family, there are metallocarboxypeptidases (MCP) that engage in the hydrolysis of peptide bonds at their C-termini.

##### 3.2.2.4. Disulfide Bond Cleaving Enzymes

Before hydrolysis by protease, the most critical stage in feather keratin degradation is the cleavage of disulfide bonds. This is because disulfide bond, particularly those that crosslink the polypeptide chains, acts as a barrier preventing keratin from being hydrolyzed by proteases. Enzyme activity is considerably decreased, or catalysis becomes difficult to occur, when the disulfide bond remains present in the process. Enzymes such as disulfide reductase and glutathione reductase can directly cleave the disulfide bond, while cysteine dioxygenase contributes indirectly by generating sulfite, a chemical reductant that breaks disulfide bonds. Most studies have reported that combining keratinolytic enzyme with disulfide reductase improves rate of keratin breakdown or increases enzyme activity. Rahayu et al. [115] showed that the coupling of purified keratinase and purified disulfide reductase led to the substantial increase in enzyme activity on natural keratin substrates (feather and wool), in comparison with the activity of each enzyme alone.

Some microorganisms can degrade keratin without the need for disulfide-reducing enzymes, as they possess inherent degradation capabilities. However, the reduction of cysteine bridges can accelerate keratin degradation. This is evidenced by the fact that mixing of the two enzyme fractions, protease and disulfide-reducing enzymes, increased keratinolytic activity by more than 50-folds. In the absence of disulfide reductase, protease increased keratin degradation by about 2-folds [8].

##### 3.2.2.5. Chemicals Related to Keratin Degradation

While the disulfide bond in the keratin structure prevents the hydrolysis of keratin into amino acids and soluble peptides, the addition of a reducing agent can help keratinolytic activity. This augmentation offers supplementary catalytic sites for keratinase to facilitate catalysis. The inclusion of reducing agents such as DTT, along with surfactants including Tween-80, Triton X-100, and CTAB [28], can break the disulfide bond and improve the hydrolysis of keratin significantly [116].

## 4. Purification and Cloning of Keratinolytic Enzymes

Keratin degradation necessitates several keratinolytic enzymes to accomplish the degradation process. Investigating the mode of action of individual enzyme(s) responsible for the breakdown and the resulting product generation from the specific cleavage of compact substrate keratin holds particular significance. Furthermore, comprehending the biochemical properties of the individual enzymes is crucial. This includes determining their temperature and pH optimum, assessing thermal and acid/alkali stability, and investigating the role of metal ions and inhibitors on enzyme activity regulation and substrates specificity. This is essential because these variables directly affect enzyme activity. Consequently, identifying optimal conditions for enzyme activity becomes imperative when selecting the suitable industrial process.

Purification of keratinolytic enzymes is a challenging procedure, demanding several steps and technologies to achieve an adequate amount of protein with a high purity level or even no impurity.  Table 4 shows several keratinase enzyme purification processes and their enzyme properties. Commonly, the keratinase purification strategy involves steps such as ammonium sulfate precipitation (salting in) and dialysis (salting out), ultrafiltration (UF), gel filtration chromatography, and ion exchange chromatography.

Ammonium sulfate precipitation serves as a common method for retrieving proteins from a solution. Typically, an ammonium sulfate concentration ranging from 20% to 80% (saturation) is selected to precipitate the enzyme from the culture supernatant, as shown in Table 4. Proteins in solution create hydrogen bonds with water by exposing their polar and ionic side chains. Numerous small, highly charged ions, such as ammonium sulfate, compete with the proteins for water molecule binding. The elimination of water molecules from the protein leads to precipitation, which diminishes the protein's solubility. The amount and position of polar groups, the protein's molecular weight, the solution's pH, and the temperature during precipitation are crucial factors affecting the concentration at which a protein can precipitate. Following precipitation, the precipitated enzyme is subjected to dialysis against buffer for salt removal. For example, precipitating crude keratinase from *Streptomyces* sp. with 50% ammonium sulfate, followed by dialysis, resulting in a yield of 94.5% and a purification fold of 1.2 [118]. The culture supernatant containing keratinolytic activity from *Meiothermus* sp. I40 was subjected to precipitation using ammonium sulfate saturation ranging from 20% to 80%, resulting in a 91.7% activity recovery rate and a purification fold of 2.5 [117].

UF is a membrane filtration technique in which water is pushed through a semipermeable membrane by hydrostatic pressure. The UF process is utilized to eliminate or remove particles, bacteria, viruses, toxins, and other pathogens, yielding a liquid with high purity and low salt density. While the chemical, molecular, and electrostatic characteristics of the sample can affect filter permeability, the primary factor driving separation is molecule size. Due to the MW cutoff values, UF can only gather molecules that have size differences of at least an order of magnitude (ranging from 1 kDa to 1000 kDa). UF membranes can be employed to purify or collect the filtrate. Generally, the membrane used for UF is made of cellulose matrices, whose MW cutoff ranges from 5 to 30 kDa MW cutoff values. For example, the crude keratinase from *B. megaterium* MS941 was concentrated using 10 kDa MW cutoff UF. This process resulted in an increase in specific enzyme activity from 15.0 to 34.4 U/mg protein [119].

Ion exchange chromatography is employed to separate a mixture of proteins into their constituent components by leveraging their interactions with an ion functional group present on an inert matrix. Ion exchange chromatography, also referred to as ion chromatography, is a separation technique utilizing ion exchangers to separate ions and polar molecules. The underlying principle involves the reversible exchange of ions between the target ions in the sample (protein) solution and the ions immobilized on the ion exchangers.

Cationic (e.g., CM- and SP-Sepharose) and anionic (e.g., DEAE- and Q-Sepharose) exchangers are two types of exchangers applicable for protein purification. Cationic exchangers possess a negatively charged group that attracts positively charged ions. These exchangers are also recognized as acidic ion exchange materials, as their negative charges originate from the ionization of an acidic group. Anionic exchangers possess positively charged groups that attract negatively charged anions.

As presented in Table 4, it is observed that anion or cation exchange chromatography is commonly employed in the second or third steps of keratinase purification. Frequently, they are utilized following like ammonium sulfate precipitation or UF. A few studies applied ion exchange for the first purification step. For example, Yu et al. [124] introduced the crude keratinase from *Trichophyton mentagrophytes* to anion DEAE-cellulose column, resulting in a recovery rate of enzyme activity at 111.5% and a specific activity of 4.02 KU/mg protein. When introducing the crude keratinase from *B. subtilis* to a cationic CM-Sepharose column, a 2.2% activity yield was obtained, accompanied by a specific activity increase from 748.3 to 915 U/mg protein [120].

Gel filtration is also recognized as size-exclusion chromatography or molecular-sieve chromatography. During this process, the protein sample, varying in molecular size, permeates the pores of the gel filtration medium. The stationary phase in this method consists of beads of hydrated sponge-like materials, featuring pores with a small molecular diameter.

When a sample solution containing protein molecules of various sizes is passed through a column with molecular sieves, protein molecules larger than the pores in the filtration medium travel quickly through the column. Smaller protein molecules pass through the pores in the gel and move slowly through the column. They are eluted in a sequence of decreasing molecular size. The molecular mass of the smallest molecule unable to permeate the gel's pores is known as the gel's exclusion limit. However, as this technique separates protein molecules based on size, it maintains the native form of keratinase. For example, purifying keratinase from the crude enzyme from *B. subtilis* KD-N-2 using Sephadex G-75 increased the specific activity from 5 to 13 U/mg protein [73].

The advancements in genome sequencing techniques, coupled with the accessibility of genomic data through online databases, have significantly enhanced the capabilities of molecular cloning and gene manipulation. These methodologies have emerged as valuable tools for the overproduction of target proteins and the purification of specific enzymes from microbial enzyme preparations. This progress emphasizes the pivotal role of cutting-edge biotechnological approaches in facilitating the detailed study and utilization of microbial enzymes for various applications. The target keratinase gene can be cloned into vectors for overproducing recombinant keratinase. Rather than natural hosts, *Escherichia coli* host system is often employed for both cloning and expressing recombinant protein. Indeed, the proteins produced by *E. coli* can be either intracellular or extracellular. With the designed His-tag connecting with the target protein, the resulting fusion allows the His-tag fused keratinase strongly bind to the Ni^2+^‐NTA column. This high binding affinity simplifies purification process and reduces contamination from proteins present in the culture medium. For example, the gene (kerT1) is responsible for encoding a putative keratinase from *Thermoactinomyces* sp. YT06 was cloned and expressed in *E. coli* BL21(DE3), resulting in a purified recombinant keratinase with a yield of 39.16%. This process achieved a 65.27-fold purification, yielding a specific activity of 1325 U/mg [83]. The keratinase gene from *Geobacillus stearothermophilus* AD-11 was cloned and expressed in the same *E. coli* BL21(DE3) host. This process resulted in a 61.2% recovery rate, with a specific activity of 1437.6 U/mg protein and a purification fold of 6.2 [103]. The high yield of protein and activity is due to the overproduction of target protein in the modified *E. coli* BL21 expression host system.

Aside from keratinase overexpression, gene manipulation techniques such as site-directed mutagenesis (SDM) and random-and-extensive mutagenesis (REM) are employed to improve keratinase activity, thermal stability, and feather degradation potential [40, 128–131]. For example, Fang et al. [128] reported that the mutants produced keratinase with good stability at 70°C, high substrate specificity, improved enzyme secretion, and enhanced catalytic activity by over 30%, compared to the wild type.

## 5. Bioconversion of Chicken Feathers and the Use of Keratinolytic Enzymes and Microbes in Industry

**Figure 4:**
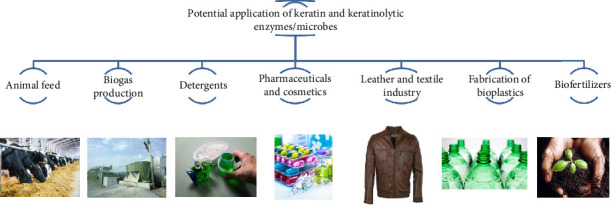
Advancements in biotechnological applications of keratin and keratinolytic enzymes.

Utilizing keratinolytic enzymes and microorganisms for biological conversion, chicken feathers can be transformed into a range of value-added products. Chicken feathers can serve as a starting material for keratinolytic enzymes and microorganisms in several industries, including animal feed, biogas production, detergents, pharmaceuticals, leather, textile industries, bioplastics manufacturing, biofertilizer production, and others (Figure 4).

### 5.1. Animal Feed

Traditionally, chicken feathers were pulverized into powder and used as animal feed. However, animals cannot obtain nutrients from chicken feathers due to their complex structure, which is challenging for enzymes within the body to break down. Therefore, hydrolyzate after feather degradation by keratinolytic enzymes is of interest because this hydrolyzate contains both essential and nonessential amino acids, which can be directly absorbed by animals [132, 133].

Deivasigamani and Alagappan [134] employed *Bacillus* sp. to convert chicken feather waste into crude protein content (1.44 mg/mL) within 5-day period. This crude protein had a high nutritional value and could potentially serve as feed for cattle or fish. Moreover, the inclusion of *B. licheniformis* PWD-1 keratinase in the diet led to an improved feed conversion ratio, while its supplementation in starter diets increased broiler chicken growth at 22 and 27 days of age. Furthermore, treating the meal with the enzyme at various dosages resulted in reduced jejunal viscosity [93]. Furthermore, chickens that were provided a diet enriched with *B. licheniformis* PWD-1 hydrolyzate, abundant in free amino acids, grew faster, similar to chickens that received a diet supplemented with soybean meal [135]. In addition, *B. subtilis* RSE163 demonstrated the ability to degrade chicken feather substrates, reaching a peak keratinase production of 366 ± 15.79 U. Moreover, the concentration of the crude hydrolyzate at 0.015% showed no cytotoxicity on the liver cell line (HepG2). This highlights the potential use of *B. subtilis* RSE163 in producing digestible animal feed rich useful amino acids and peptides [136].

### 5.2. Biogas Production

Currently, fossil fuels meet approximately 80% of the world's energy consumption. These resources are limited, and rising fuel costs hasten the need to substitute fossil fuels with renewable, environmentally friendly alternatives [137]. Biogas, particularly methane, serves as a gaseous biofuel derived from anaerobic digestion of organic material. The process of biogas production is complexed, involving various steps such as hydrolysis, acetogenesis, acidogenesis, and methanogenesis.

Various organic materials, such as agricultural wastes, sludge, animal by-products (e.g., manure, bone and meat meal, and fish waste), can serve as substrates for biogas production [138–140]. Keratin-based materials such as wool and chicken feathers have shown promise as feedstock for biogas production, attributed to the presence of useful amino acids [141].

As per the literature [142–144], various forms of chicken feathers have been investigated as biogas production substrates. These include untreated feather (chopped feather), feather hydrolyzate, feather broth (hydrolyzate with keratinolytic bacteria), and chemically/thermally pretreated feather (soluble form). However, the yield of biogas production through the anaerobic digestion of native chicken feathers is comparatively low [141–143]. This is attributed to the recalcitrant nature of keratin-rich materials, possibly posing difficulty in degradation to microbial activity in the anaerobic digestion system [145].

Furthermore, keratin cannot be degraded by common proteases [95], and within anaerobic digestion systems, keratinolytic activity might be constrained due to scarcity or lack of microorganisms that produce keratin-specific proteases [145]. This hydrolytic activity holds importance for the decomposition of keratin-rich organic materials during the hydrolysis stage, which marks the initial step of anaerobic digestion. This process renders the organic substrate accessible and viable for the anaerobic microbiota, expediting methane production [146, 147].

Although the protein degradation in general is not a limiting step in common anaerobic digestion, the increased decomposition of hard-to-degrade protein substrate, such as keratin, could potentially enhance the efficiency of the whole biogas production process [148]. Therefore, it is recommended that chicken feather should be pretreated/solubilized, rendering them amendable to enzymatic action before their utilization in the hydrolysis stage. This approach aims to enhance the yield of biogas production [141, 142, 144].

Utilizing keratinolytic enzymes and bacteria for biological pretreatment represents a viable approach to deconstruct the feather keratin, thereby releasing amino acids and short peptides for further microbial biogas fermentation processes [149]. For example, the addition of alkaline endopeptidase enzyme at a rate of 0.53 mL/g volatile solids to chicken feathers as enzymatic pretreatment resulted in the methane yield increase from 0.18 to 0.40 Nm^3^/kg. This corresponds to 122% improvement compared to the yield obtained from feathers without enzyme supplementation [141]. Furthermore, the enzymatic pretreatment rendered the feather more amenable to microbial activity compared to the thermal pretreatment using autoclave at 120°C for 10 min. Using the thermal method, methane yield witnessed an approximate 11% increment in comparison with the untreated one, following anaerobic digestion at 55°C for a span of 50 days [141].

The application of α- and β-keratinases sourced from *Bacillus* sp. C4 was explored as an alternative pretreatment technique for chicken feather fermentation. The resultant feather hydrolyzate (liquid fraction only) was tested for biogas production, employing either anaerobic sludge or bacteria granules as inoculums [144]. In comparison with untreated feathers, biogas fermentation of feather hydrolyzate with anaerobic sludge and bacterial granules led to methane outputs that were enhanced by 292% and 105%, respectively. This result suggests that untreated feathers can be used as a substrate for anaerobic digestion, when utilizing both anaerobic sludge and bacteria granules as inoculums. However, *Bacillu*s sp. C4–treated feather hydrolyzate gave better fermentation performance and methane yields, even when utilizing the same inoculums employed in the anaerobic digestion process [144].

An alternative approach to improve methane production from chicken feathers is codigestion, which entails anaerobically digesting multiple substrates together. This strategy aims to optimize nutrient balance and microbial synergy through the utilization of different organic matters [149]. Several animal by-products have been reported as codigestion substrates alongside chicken feathers to enhance methane yield. Such by-products include horse dung [143], swine manure [149], slaughterhouse sludge, and dairy manure [150]. Studies have reported a substantial enhancement in methane production (0.16–0.19 L CH4/g VS_initial_) through the codigestion of microbially treated feather hydrolyzate with matured swine manure (static incubation for 90 days). Conversely, cosubstrate between untreated feather and matured swine manure or horse dung showed negative effect, resulting in reductions of methane yields by 15%–25% and 14.84%, respectively [143, 149]. This superior effect can likely be attributed to slow degradation.

In contrast, when fresh swine manure was codigested with feather hydrolyzate at high concentration (6.8% total solid), the methane yield showed a 43% reduction owing to the ammonia inhibition [149]. Therefore, it is reasonable to assume that microbial-treated feather hydrolyzate could increase methane production through better biodegradation. High ammonia levels caused by microbial activity during anaerobic codigestion may reduce methane output at high concentrations. Hence, research into the creation of biogas from keratin is still in its early stages, necessitating further study on fermentation mechanisms.

### 5.3. Detergent

Chicken feathers hold potential as an economical growth substrate for microorganisms that produce keratinase, and furthermore, keratinase enzymes with high alkali tolerance could find application in the detergent industry. Keratinase is a versatile enzyme known to effectively disintegrate protein clots without damaging the clothes.

*Paenibacillus woosongensis* TKB2 alkaline keratinase has potential use in the laundry business, effectively removing blood stains from surgical cloths. It also demonstrates remarkable efficiency in swiftly eliminating in composite stains comprising blood, egg yolk, and chocolate, as observed in a study by Paul et al. in 2014 [151]. Due to its cost-effective keratinase production and high enzyme stability, *Arthrobacter* sp. KFS-1 has demonstrated application potential within the detergent industry. The keratinase enzyme displayed stability in the presence of various detergents including Maq, Omo, Surf, Sunlight, and Ariel. However, the degree of enzyme stability appears to be influenced by differences in the formulation ingredients of these detergents, as noted by Nnolim et al. in 2020 [69].

Furthermore, keratinase has been reported to maintain remarkable stability in the presence of 15% Tween-80 or Triton X-100, strong anionic surfactants, particularly SDS and sulfobetaine, as well as nonionic surfactants, denaturing agents, anionic surfactants, and bleach agents [87]. These findings lend support to the viability of incorporating it as a cleansing ingredient in detergent formulations.

### 5.4. Pharmaceutical and Cosmetic Industries

Numerous topical treatments for nails, calluses, acne, scars, prions, and skin use keratinase as an active component. Additionally, keratinase could be found in the pharmaceutical sector to facilitate medication administration through nail plates. Certainly, the primary drawback of topical treatment lies in the permeation of medicine through the nail plates, which is the root cause of recurrent onychomycosis infections.

Chemical therapy for onychomycosis has a bad smell and needs to be given in very high dosages. Because keratins constitute a major portion of the nail plates, keratinases can be used to successfully treat this ailment [152]. For example, keratinase KerN, a subtilisin-γ-glutamyl transpeptidase complex, has been utilized to increase medication administration through nails and improve drug delivery via nails [152]. Also, due to its specific affinity for keratin substrate, keratinase holds promise for use in dehairing cream without causing skin irritation in humans [153, 154].

Bioactive peptides emerge as a result of keratin degradation, and these peptides have therapeutic use as antioxidants and antityrosinase substances [155] (Table 5). Evidence has demonstrated the efficacy of keratin-derived molecules in inhibiting a wide range of pathogenic bacteria, encompassing both gram-negative and gram-positive [32, 155, 165, 166].

Keratin peptides with a molecular weight below 3 kDa, for instant, inhibited the growth of *E. coli* when generated through an instant catapult steam explosion approach. This inhibition was attributed to the elevated concentration of hydrophobic residues in keratin peptides [32].

Keratin hydrolyzate possesses anti-inflammatory characteristics, suppresses amyloid aggregation during initial stages [167], and showcases antiaging properties [168]. Research indicates that keratin peptide with a low molecular weight (<1 kDa), obtained through anaerobic digestion of chicken feathers by *Fervidobacterium islandicum* AW-1, contributed to enhanced skin health. This was achieved by suppressing the expression of UVB-induced MMP-1 and MMP-13, both of which play a pivotal role in human dermal fibroblast-related skin aging [169].

This result suggests that feather keratin peptides are attractive candidates for cosmeceutical products such as antiaging creams. Furthermore, keratin peptides find application in hand care products to hydrate dry hand skin and increase elasticity, owing to their substantial water-retention capability [170]. Moreover, they have the ability to retain moisture, mitigating skin irritation and the damaging effects of sodium lauryl sulfate [170].

### 5.5. Leather and Textile

Unhairing represents a necessary yet hazardous step in the leather industry. In earlier years, the conventional method of employing inorganic sulfide treatment was extensively utilized for depilation, despite its adverse effects on leather quality and environmental pollution. Biocatalysts such as keratinases have been employed in lieu of inorganic sulfide, which has greatly improved the efficiency of the dehairing process and provided substantial environmental benefits. During leather processing, the pH of liming and unhairing is typically basic; thus, alkaline keratinases are required. Research reported that, in dehairing goat skin, keratinases from *Acinetobacter* sp. were able to clean and smooth the surface of skin to a greater extent than chemically treated skin [81].

The evaluation of crust quality aligned closely with findings from other studies, highlighting that enzymatic treatment of leather crust can yield extremely excellent quality in contrast to chemically treated leather crust [171]. Moreover, the collagen matrix of the skin was not affected by the enzymatic treatment, possibly due to the minimal collagenolytic and elastinolytic activity exhibited by keratinase [123, 172]. This is likely due to the absence of sodium chloride or sulfuric acid usage. Enzymatic dehairing holds the potential to minimize chemical consumption and by-product generation, thereby lowering the toxicity of wastewater [173].

### 5.6. Production of Bioplastic

Plastics are currently employed across a broad spectrum of applications, encompassing food packaging and healthcare products. Chicken feather-derived keratin protein finds diverse applications in the manufacturing of films, sponges, and fibers, either independently or in combination with synthetic and natural polymers [174]. Plastics originating fossil pose environmental risks and are resistant to natural degradation. Alshehri et al. [175] cultivated *B. cereus* BAM3 on chicken feather-based medium, utilizing it as a keratin source for bioplastic production. The extracted keratin, when combined with 2% (w/v) glycerol as a plasticizer, demonstrated good mechanical characteristics. Furthermore, the keratin/glycerol plastic film displayed uniform morphologies without any holes, voids, or edges, and exhibited notable thermal stability. Moreover, the keratin-based film was completely degraded by proteases within 11 h, providing clear evidence of its biodegradability [53].

### 5.7. Biofertilizer

Although chemical fertilizers are commonly used, their utilization holds the potential to completely ruin and devastate the environment. Hence, biofertilizer is an alternative to chemical fertilizer due to its minimal environmental impact and its ability to contribute to various environmental advantages.

The fermented hydrolyzate that underwent filter sterilization, for instance, exhibited the ability to significantly enhance both seed germination and seedling growth of Bengal gram (*Cicer arietinum*). Additionally, it increased soil fertility by augmenting N, P, K, and the C/N ratio by 1.2 times, while also leading to a threefold promotion in nodule formation. In comparison with the control soil, this led to a twofold increase in free-living nitrogen fixers and 5.8-fold rise in phosphate solubilizers [176]. Chicken feather hydrolyzates can serve as an economical liquid organic fertilizer source. The application of hydrolyzate has the potential to enhance the length and growth of Bengal gram seedlings, along with fostering an increase in the soil microbial population [177]. Furthermore, the application of feather hydrolyzate to soil resulted in the stimulation of plant growth through seed germination media, attributed to the production of indole-3-acetic acid [178].

## 6. Conclusion

In most cases, keratin by-products have little to no economic value and can even be harmful to the natural realm. Improperly disposed of keratin waste can lead to disease transmission and emit a wide range of toxins. The keratin bioconversion technique finds applications across diverse industries, encompassing agriculture, cosmetics, pharmaceuticals, biotechnology, and the delivery of medicine. The utilization of keratinolytic enzymes and microorganisms offers several advantages over chemical methods, including less pollution and no transformation of the product into a potentially dangerous substance. We are of the opinion that the biological conversion of keratin waste into valuable products and the utilization of keratinase in the biotechnological sector not only contribute to environmental preservation but also generate economic benefits for industries and society. This includes bolstering income, productivity, and the inclusion of marginalized groups, aligning with the objectives of the SDGs.

## Figures and Tables

**Table 1:** Amino acid composition in chicken feathers [39].

Amino acid	Quantities
*Nonpolar amino acid*	
Ala	8.7^∗^
Gly	13.7^∗^
Ile	3.2^∗^
Leu	8.3^∗^
Pro	9.8^∗^
Val	7.8^∗^
Phe	3.1^∗^
Tyr	1.4^∗^
Total	56^∗^

*Acidic/basic and polar amino acid*	
Asp	5.6^∗^
Glu	6.9^∗^
Arg	3.8^∗^
His	0.2^∗^
Lys	0.6^∗^
Total	17.1^∗^

*Neutral and polar amino acid*	
Ser	14.1^∗^
Thr	4.1^∗^
Cys	7.8^∗^
Met	0.1^∗^
Total	26.1^∗^

⁣^∗^Unit, g of amino acid/100 g of proteins.

**Table 2:** Extraction of keratins from chicken feathers by chemical methods.

No.	Chemical	Substrate	Part of feather	Substrate loading (%)	Temperature (°C)	Time (h)	Keratin yield (%)	Reference
1	1.66 M 2-mercaptoethanol	Chicken feathers	Whole feather (calamus/rachis [β-sheet] and barbs/barbules [α-helix])	4	50	2	83.8	[48]
2	0.5 M sodium bisulfite	Chicken feathers	Whole feather (calamus/rachis [β-sheet] and barbs/barbules [α-helix])	4	50	2	82.4	[48]
3	2.5 M NaOH	Chicken feathers	Whole feather (calamus/rachis [β-sheet], and barbs/barbules α-helix])	4	70	1.15	93.7	[48]
4	1.4 M 2-mercaptoethanol	Chicken feathers	Whole feather (calamus/rachis [β-sheet] and barbs/barbules [α-helix])	4	40	0.5	75	[50]
5	50 g/L sodium sulfite	Chicken feathers	Whole feather (calamus/rachis [β-sheet] and barbs/barbules [α-helix])	10	30	24	55	[51]
6	Ionic liquid [HOEMIm][NTf2]	Chicken feathers	Whole feather (calamus/rachis [β-sheet] and barbs/barbules [α-helix])	2.5	80	4	21.5	[52]
7	0.5 mol/L sodium sulfide	Chicken feathers	Whole feather (calamus/rachis [β-sheet] and barbs/barbules [α-helix])	5	40	6	∼88	[47]
8	0.165 mol/L L-cysteine	Chicken feathers	Whole feather (calamus/rachis [β-sheet] and barbs/barbules [α-helix])	5	40	6	∼66	[47]
9	0.2 M sodium sulfide	Chicken feathers	Whole feather (calamus/rachis [β-sheet] and barbs/barbules [α-helix])	2.5	60	5	—	[53]
10	1.78% NaOH and 0.50% NaHSO_3_	Chicken feathers	Whole feather (calamus/rachis [β-sheet] and barbs/barbules [α-helix])	20	87	∼2	68.29	[54]

**Table 3:** Biochemical property of keratinases from different microbial sources.

No.	Species	Source	Enzyme	Growth substrate loading (%, w/v)	Growth substrate used in fermentation	Substrate tested for keratinase activity	Substrate tested for proteolytic enzyme activity	Keratinase activity					Degradation (%)	Reference
								pH optimum	pH stability	Optimal temperature (°C)	Temperature stability (°C)	The highest activity (U/mL)		
1	*Bacillus licheniformis* N22	Farmyard wastes	Keratinase	1.1	Chicken feather	Keratin azure	Casein	8.5	6–12	50	—	11.0 ± 0.71	—	[64]
2	*Bacillus licheniformis* WHU	Farmyard wastes	Keratinase KerBL	—	Chicken feathers	Keratin azure	Azocasein	10.5	6–11.5	65	40–75	23.005 ± 1.8	—	[65]
3	*Acidophilic Bacillus* sp. Okoh-K1	Municipal waste	Alkaline metallokeratinase	0.1	Chicken feathers	Keratin azure	—	8	6.0–9.0	60	50–60	2350.09 ± 107.99	—	[66]
4	*Bacillus* sp. NKSP-7	Poultry dump soil	Keratinase	1	Chicken feathers	Keratin azure	—	7.5	5.5–9.5	65	20–60	51.5	89.51	[67]
5	*Bacillus pumilus* AR57	Poultry soil wastes	Alkaline serine keratinase	1	Chicken feathers	Keratin azure	—	9	—	45	—	—	100	[24]
6	*Bacillus thuringiensis* MT1	Cattle -yard	Keratinase	1	Donkey hair	Keratin powder	—	9	7–11	50	20–80	422	95	[68]
7	*Arthrobacter* sp. KFS-1	Dump site	Alkaline keratinase	0.25	Chicken feather	Keratin azure	—	8	6.0–9.0	60	50–60	1559.09 ± 29.57	—	[69]
8	*Bacillus subtilis* NRC 3	Poultry soil wastes	Metallokeratinase	0.5	Chicken feather	Keratin azure	Azocasein	8	5–10	50	20–60	5233	—	[70]
9	*Bacillus* sp. P7	Intestines of the teleost fish Jaraqui	Keratinase	1	Chicken feathers	Keratin azure	Azocasein	9	8–12	55	30–80	12,966.6	—	[71]
10	*Bacillus subtilis* DP1	Poultry farm soil	Alkaline keratinase	2	—	Ground chicken feather	—	10	8–12	37	20–50	379.65	—	[72]
11	*Bacillus subtilis* KD-N2	Local poultry plant	Keratinase	1	Chicken feather	Keratin azure	—	8.5	—	55	40–70	63.3	—	[73]
12	*Pseudomonas* sp., MS21	Poultry farm soil	Keratinase	1	Chicken feather	Keratin azure	—	8	—	37	—	40	—	[74]
13	*Bacillus zhangzhouensis*	Poultry feather	Keratinase	0.1	Chicken feather	Azokeratin	—	9.5	7–10.5	60	30–70	49.96	—	[28]
14	*Stenotrophomonas maltophilia* DHHJ	Poultry decomposition feathers	KeratinaseL1	2	Chicken feather	Keratin powder	—	7.8	5.0–9.0	40	20–40	32	—	[75]
15	*Stenotrophomonas* sp. D-1	Deer Fur from zoo	Keratinase PD-1	1	—	Keratin powder (mainly from human hair)	Azocasein	8.0 and 9.0	7.0–10.0	40	30–40	160	—	[76]
16	*S. maltophili*a BBE11-1	Local poultry farm	Keratinase K1	1	Chicken feather	Feather powder	Casein	9	7.0–11.0	50	—	126 ± 5.2	—	[77]
17	*S. maltophilia* BBE11-1	Local poultry farm	Keratinase K2	1	Chicken feather	Feather powder	Casein	9	7.0–11.0	50	—	24 ± 3.4	—	[77]
18	*Bacillus licheniformis.*	Soil	Keratinase	1	Chicken feather	Keratin azure	—	8.5	7.0–9.0	60	—	3.67	—	[78]
19	*Paecilomyces marquandii*	—	Keratinase	1	Keratin powder	Keratin powder (stratum corneum of the human sole)	—	8	6.0–11.0	65	—	230.6	—	[79]
20	*Bacillus pumilus* NRC21	Soil from chicken farms	Serine metallokeratinase	0.5	Chicken feather	Keratin azure	Azocasein	7.5	5.0–10.0	50	20–60	170	—	[80]
21	*Brevibacillus parabrevis* CGMCC 10798	Rich-keratin sheepfold	Keratinase	1	Wool powder	Keratin	—	8	6.0–9.0	60	—	198	—	[81]
22	*Aspergillus flavus* K-03	Poultry farm	Keratinase	2	Chicken feather	Keratin azure	—	8	7.0–10.0	45	30–70	27.24	—	[82]
23	*Thermoactinomyces* sp. YT06	—	Recombinant keratinase (rKERTYT)	—	Chicken feather	Keratin	—	8.5	6.0–11.0	65	50–60	20.3	—	[83]
24	*Thermoactinomyces* sp. YT06	Poultry compose	Keratinase	1	Chicken feather	Soluble keratin	Casein	9	8.0–11.0	65	50–60	42	—	[84]
25	*Bacillus halodurans* PPKS-2	—	Recombinant keratinases-I	1	Chicken feather	Keratin	Casein	11	7.0–13.0	60–70	—	141	—	[85]
26	*Aspergillus clavatus* VKPM F-1593	—	Alkaline subtilisin-like nonglycosylated protease	0.5	Chicken feather	Keratin	Hammerstein casein	8	4–10	50	25–40	23	—	[86]

**Table 4:** Purification techniques used for obtaining a pure form of keratinases.

No	Microbial species	Substrate	Purification techniques	Yield (%)	Specific activity (U/mg protein)	Reference
1	*Meiothermus* sp. I40	Keratin azure	Crude enzyme	100	1169.3	[117]
			Ammonium sulfate precipitation (20%–80%)	91.7	2974.5	
			Hydroxyapatite (HPT)	72.1	6869.5	
			Superdex 200 pg	45	35,364.8	

2	*Streptomyces* sp.	Keratin azure	Crude enzyme	100	227.5	[118]
			Ammonium sulfate precipitation (0%–50%)	94.6	265	
			Sephacryl S-100	66.7	686.5	

3	*Bacillus megaterium* MS941	Keratin azure	Crude enzyme	100	15	[119]
			Ultrafiltration (10 kDa MW cutoff)	73.5	34.4	
			Affinity chromatography	9.3	1277.7	

4	*Pichia pastoris*	Keratin azure	Crude enzyme	100	5.4	[119]
			Ultrafiltration (10 kDa MW cutoff)	72.3	20.2	
			Affinity chromatography	1.4	365.7	

5	*Actinomadura keratinilytica* strain Cpt29	Keratin azure	Crude enzyme	100	1453	[87]
			Ammonium sulfate precipitation (40%–70%)	82	8213	
			Heat treatment (30 min at 80C)	41	23,875	
			Sephacryl S-200	11.6	70,000	

6	*Bacillus* subtilis RM-01	Chicken feather	Crude enzyme	100	748.3	[120]
			CM-cellulose	2.2	915.0	
			Sephacryl S-200	0.7	2843.3	
			Reverse-phase HPLC	0.3	6800.0	

7	*Bacillus* sp. NKSP-7	Keratin azure	Crude enzyme	100	12.11	[67]
			Heat treatment	79.64	24.91	
			Ammonium sulfate precipitation (75%–80%)	59.96	39.55	
			Ion exchange chromatography	22.15	462.54	

8	*Scopulariopsis brevicaulis*	Chicken feather	Crude enzyme	100	10.3	[121]
			Ammonium sulfate precipitation (80%)	47	17.4	
			DEAE-cellulose column	32	37.4	
			Sephadex G-100 chromatography	24.1	70.6	

9	*Doratomyces microsporus*	Keratin powder	Lyophilized powder	100	44.5	[122]
			Phenyl-superose	42	238.9	
			Superose-12	21.6	430.0	

10	*Bacillus* subtilis KD-N2	Keratin azure powder	Crude enzyme	100	5.0	[73]
			Sephadex G-75	12.8	13.0	
			DEAE-Sepharose FF	7	51.9	
			Sephadex G-75	4.6	63.3	

11	*Pseudomonas* sp. MS21	Keratin azure	Ammonium sulfate precipitation (40%)	100	4.86	[74]
			CM-cellulose	30	8.24	
			Sephadex-75	12	21.54	

12	*Bacillus zhangzhouensis*	Azokeratin	Crude enzyme	100	2.98	[28]
			Dialysis product	6.58	3.44	
			SP-Sepharose	1.65	39.33	
			Q-Sepharose	0.66	81.85	

13	*Bacillus pumilus* NRC21	Keratin azure	Crude enzyme	100	5439	[80]
			CM-cellulose	21.3	15,294	
			Sephadex G-100	17	230,600	

14	*Brevibacillus parabrevis* CGMCC 10798	Keratin	Crude enzyme	100	501.34	[81]
			Ammonium sulfate precipitation (40%–70%)	65.04	543.43	
			HiPrepTM DEAE FF 16/10	17.19	6605.48	

15	*Brevibacillus* sp. strain AS-S10-II	Keratin	Crude enzyme	100	12,000	[123]
			80% acetone precipitate fraction	91	105,000	
			GF-I (Brevicarnase)	58.5	216,000	

16	*Aspergillus flavus* strain K-03	Keratin azure	Crude enzyme	100	27.42	[82]
			Ammonium sulfate precipitation (80%)	51.26	65.23	
			Sephadex G-100	33.35	244.96	
			DEAE-Sephadex A-50	32.36	316.15	

17	*Thermoactinomyces* sp. YT06	Keratin	Crude enzyme	100	20.3	[83]
			Ammonium sulfate precipitation	65.02	60	
			Affinity chromatography	39.16	1325	

18	*Bacillus halodurans* PPKS-2	Keratin	Crude enzyme	100	141	[85]
			Ammonium sulfate precipitation (60%)	78.4	310	
			DEAE-Sephadex	40	790.9	
			Sephadex G-200	11.17	3039.2	

19	*Trichophyton mentagrophytes*	White Guinea pig hair	Crude enzyme	100	1.35	[124]
			DEAE-cellulose	111.5	4.02	
			CM-cellulose	39.5	6.03	
			Gel filtration	17.9	30.1	

20	*Streptomyces* sp.	Keratin azure	Crude enzyme	100	227.5	[118]
			Ammonium sulfate precipitation (50%)	94.6	265	
			Gel filtration	66.7	686.5	

21	*Microbacterium* sp.	Azocasein solution	Crude enzyme	100	59.3	[125]
			Freeze-dried	70.9	66.1	
			Sephadex G-100	52.8	1428.6	
			Q-Sepharose	34.2	15,125	

22	*Bacillus subtilis* MTCC	Horn meal	Crude enzyme	100	90.9	[126]
			Salt precipitation	85.3	363.6	
			CMC ion exchange	37.35	3727.7	
			Gel filtration	27.6	4181.8	

23	*Chryseobacterium* sp. Kr6	Keratin azure	Crude enzyme	100	168.77	[127]
			Phenyl Sepharose	1.31	552.45	
			Superose-12HR	1.18	2406.68	

**Table 5:** Radical scavenging activity of bioactive peptide derived from keratin hydrolyzate.

No.	Microbial	Substrate	Method	Radical scavenging activity (mg/mL)	Reference
1	*Bacillus* sp. RCM-SSR-102	Chicken feather	DPPH IC_50_	1.02 ± 0.01	[31]
2	*Bacillus* sp. RCM-SSR-102	Chicken feather	ABTS IC_50_	0.2	[31]
3	*Bacillus* sp. MPTK6	Chicken feather	DPPH IC_50_	0.5–3.5	[156]
4	*Bacillus* pumilus A1	Chicken feather	DPPH IC_50_	0.3	[157]
5	*Bacillus pumilus* A1	Feather	DPPH IC_50_	0.14 ± 0.01	[158]
6	*Bacillus cytotoxicus*	Chicken feather	ABTS IC_50_	0.16	[159]
7	*Chryseobacterium sediminis* RCM-SSR-7	Chicken feather	DPPH IC_50_	0.102	[160]
8	*Chryseobacterium* sp. kr6	Chicken feather	ABTS IC_50_	18.3	[161]
9	*Bacillus amyloliquefaciens* KB1	Chicken feather	DPPH IC_50_	0.7	[162]
10	*Streptomyces* sp. MAB18	Chicken feather	DPPH IC_50_	78 ± 0.28	[163]
11	*Bacillus licheniformis* BBE11-1	Chicken feather	DPPH IC_50_	0.3	[164]

## Data Availability

The data used to support the findings of this study are included within the article.
